# Comparison of emergency department utilization trends between the COVID-19 pandemic and control period

**DOI:** 10.1097/MD.0000000000026847

**Published:** 2021-08-13

**Authors:** Soo Kang, Tae Kyu Ahn, Young Ho Seo, Young Ju Suh, Jin Hui Paik

**Affiliations:** a Department of Emergency Medicine, Inha University School of Medicine, Incheon, Republic of Korea; b Department of Biomedical Sciences, Inha University School of Medicine, Incheon, Republic of Korea.

**Keywords:** COVID-19, emergency department, Korea, SARS-CoV-2

## Abstract

Infectious disease pandemics has a great impact on the use of medical facilities. The purpose of this study was to analyze the effects of coronavirus disease 2019 (COVID-19) on the use of emergency medical facilities in the Republic of Korea. This single-center, retrospective observational study was conducted in a tertiary teaching hospital located in Incheon Metropolitan City, Republic of Korea. We set the pandemic period as February 19, 2020 to April 18, 2020, and the control period was set to the same period in 2018 and 2019. All consecutive patients who visited the emergency department (ED) during the study period were included. Patients were divided into 3 groups according to age (pediatric patients, younger adult patients and older adult patients). The total number, demographics, clinical data, and diagnostic codes of ED patients were analyzed. The total number of ED patients in the pandemic period was lower than that in the control period, which was particularly pronounced for pediatric patients. The proportion of patients who used the 119 ambulances increased in all 3 groups (*P* *=* .002, *P* < .001, and *P* = .001), whereas the proportion of patients who visited on foot was decreased (*P* *=* .006, *P* < .001, and *P* = .027). In terms of diagnostic codes, a significant decrease was observed in the proportion of certain infectious or parasitic diseases (A00-B99), and respiratory diseases (J00-J99) in the pediatric and younger adult patient groups (*P* < .001 and *P* < .001, respectively). The COVID-19 pandemic reduced the number of ED patients; however, the proportion of patients using ambulances increased. In particular, the proportion of patients with diagnostic codes for infectious and respiratory diseases significantly decreased during the pandemic period.

## 1 Introduction

The infectious respiratory disease, known as coronavirus disease 2019 (COVID-19), occurs as a result of an infection by severe acute respiratory syndrome coronavirus 2 (SARS-CoV-2).^[1]^ This respiratory virus first appeared in Wuhan, China in 2019, and caused the current global epidemic.^[1]^ On March 12, 2020, the World Health Organization (WHO) declared a COVID-19 pandemic, and as of July 1, 2020, the impact of SARS-CoV-2 has been observed in over 200 countries, and the total number of infected patients has reached more than 10 million.^[2,3]^

The epidemic of infectious diseases causes various social impacts, especially the use of medical institutions. This was demonstrated in the past during severe acute respiratory syndrome (SARS) and novel influenza A (influenza H1N1) outbreaks.^[4–6]^ Several studies have reported that COVID-19, which is currently causing a global pandemic, has also resulted in changes in the use of emergency medical facilities.^[2,7–9]^

In 2015, the Republic of Korea experienced an infectious disease through an outbreak of the Middle East respiratory syndrome (MERS). There were many cases of transmission within medical institutions, and emergency departments (ED) were considered dangerous places for disease transmission.^[10,11]^ Consequently, the MERS outbreak had a significant impact on the use of emergency medical facilities in the Republic of Korea, and many studies have been conducted.^[12–14]^

The first case of COVID-19 in the Republic of Korea was confirmed on January 20, 2020. On February 19, due to a certain religious group, the number of COVID-19 patients had surged and continued to increase since then.^[15]^ After the MERS outbreak in 2015, there was a serious concern in the Republic of Korea regarding the risk of transmission in emergency medical facilities. Therefore, we hypothesized that the COVID-19 pandemic would have a significant impact on ED utilization trends. To verify our hypothesis, we analyzed the number and characteristics of patients who visited the ED during the COVID-19 pandemic.

## 2 Methods

### 2.1 Study population and setting

This single-center, retrospective observational study was conducted at the Inha University Hospital. This hospital is a tertiary teaching hospital located in Incheon Metropolitan City, Republic of Korea, and receives patients from the city and its surrounding region. The ED of this hospital is divided into an adult treatment area (≥15-years) and a pediatric treatment area (<15-years), and approximately 55,000 patients visit the ED annually. As this hospital was not a dedicated hospital for COVID-19, it accommodated all patients even during the pandemic. In addition, patients diagnosed with COVID-19 at other hospitals and public health centers were admitted to the isolation ward without ED visits. We included all consecutive patients who visited the ED during the study period. Patients were classified into 3 groups according to age, and those under 15 years were defined as “pediatric patients”, 15 to 60 years old as “younger adult patients”, and those over 60 years old as “older adult patients”. We also set up the “pandemic period” and the “control period” to analyze the effect of the COVID-19 pandemic on ED utilization. While the “pandemic period” was defined as the 2 months following from February 19, 2020, when the number of COVID-19 patients in this nation surged, the “control period” was defined as the same period in 2018 and 2019.

### 2.2 Data collection

The demographic and clinical data of the patients were collected from the electronic medical records. The number of patients, age, sex, patient acuity, reason for visit, means of visit, number of patients transferred from another hospital, outcome, emergency department length of stay (EDLOS), and diagnostic codes were included in the collected data.

Patient acuity classification was performed using the Korean Triage and Acuity Scale (KTAS) as a triage tool. The KTAS is a triage tool developed by the Korean Society of Emergency Medicine based on Canadian Triage and Acuity Scale, which has excellent reliability and validity in predicting the severity of patients.^[16–18]^ According to the KTAS, patient acuity was classified into 5 levels (level 1, resuscitation; level 2, emergent; level 3, urgent; level 4, less urgent; and level 5, non-urgent). We categorized these 5 levels into high acuity (KTAS levels 1, 2, or 3) and low acuity (KTAS levels 4 or 5). To minimize triage error, only qualified personnel who completed the relevant training performed the KTAS classification. The emergency medical call number in the Republic of Korea is “119” and the 119 ambulance can be requested by a telephone from homes or public facilities that are not medical institutions. However, the 119 ambulance cannot be used to transfer inpatients to higher-level medical institutions, so other ambulances must be used. Consequently, the means of visit were classified into 119 ambulances, other ambulances, and on foot. Transfer-in was defined as patients who were transferred from another hospital. Additionally, the diagnostic codes were classified and recorded according to the Korean Standard Classification of Disease-7. We reviewed the diagnostic codes of ED patients during the control and pandemic periods.

### 2.3 Statistical analysis

Categorical variables consisting of 2 categories were analyzed using the Pearson chi-square test or Fisher's exact test, while categorical variables consisting of 3 or more categories were analyzed using Fisher's exact test with the permutation resampling method for multiple testing adjustment, and the results were presented as numbers and percentages. For continuous variables, the Shapiro–Wilk normality test was performed. Variables with normal distribution were compared using Student's *t* test and presented as the means and standard deviations; those without normal distribution were compared using the Mann–Whitney *U* test and presented as median and interquartile range (IQR). *P* value <.05 was considered statistically significant. Data analysis was conducted using SAS (version 9.4; SAS Institute, Inc., Cary, NC).

### 2.4 Ethics statement

The present study protocol was reviewed and approved by the Institutional Review Board of Inha University College of Medicine (2020-07-014-000). Informed consent was waived, and the study was conducted in compliance with the principles of the Declaration of Helsinki.

## 3 Results

**Figure 1:**
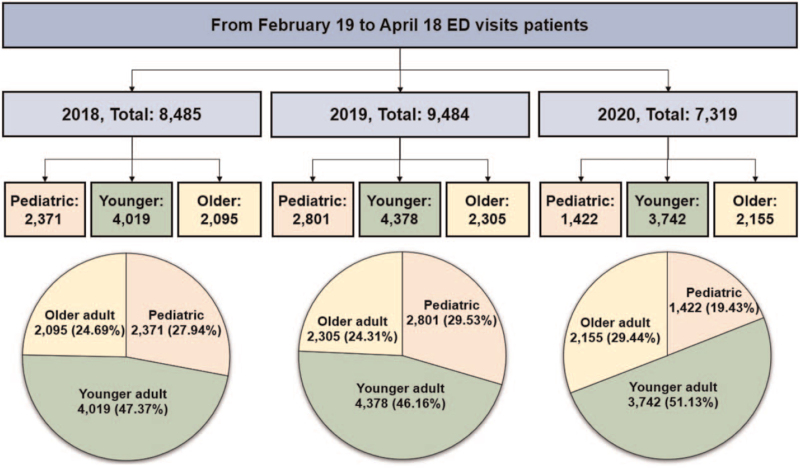
Flow chart and diagrams of emergency department patients in 2018, 2019 and 2020.

The total number of ED patients during the pandemic period was 7319, and during the control period was 8485 and 9484 in 2018 and 2019, respectively. The number of pediatric patients during the pandemic period was 1422, and during the control period was 2371 and 2801 in 2018 and 2019, respectively. The number of younger adult patients during the pandemic period was 3742, and during the control period was 4019 and 4378 in 2018 and 2019, respectively. Additionally, the number of older adult patients during the pandemic period was 2155, and during the control period was 2095 and 2305 in 2018 and 2019, respectively (Fig. 1).

### 3.1 Proportion of patients by age

Table 1 shows the changes in the proportion of patients with age. The total number of ED patients during the control period was 17,969, and 5172 were pediatric patients. Moreover, the total number of ED patients during the pandemic period was 7319, and 1422 were pediatric patients. The proportion of pediatric patients decreased during the pandemic period (19.43%) compared to the control period (28.78%). By comparison, the proportion of younger adult patients and older adult patients increased during the pandemic period (51.13% and 29.44%, respectively) compared to the control period (46.73% and 24.49%, respectively)

**Table 1:** Total number of emergency department patients during the control and pandemic periods.

Classification	Control period^∗^ (n = 17,969)	Pandemic period^†^ (n = 7319)	*P* value^‡^
Pediatric patients (<15 years old)	5172 (28.78)	1422 (19.43)	<.001
Younger adult patients (15–60 years old)	8397 (46.73)	3742 (51.13)	<.001
Older adult patients (>60 years old)	4400 (24.49)	2155 (29.44)	<.001

### 3.2 Demographic and clinical characteristics

The demographic and clinical characteristics of pediatric patients are summarized in Table 2. Age and sex were similar between the 2 periods. However, the patient acuity demonstrated a difference between the 2 periods, with an increase in the proportion of low acuity (KTAS 4, 5) during the pandemic period (74.12%) compared to the control period (65.72%, *P* < .001). Moreover, the proportion of trauma patients increased during the pandemic period (45.64%) compared to the control period (30.68%, *P* < .001). In terms of the means of visit, the proportion of patients who using the 119 ambulances increased during the pandemic period (8.93%) compared to the control period (6.38%, *P* = .002), while the proportion of patients who visited on foot decreased during the pandemic period (90.65%) compared to the control period (93.04%, *P* *=* .006). The EDLOS in the control and pandemic periods were 124 minutes (IQR, 67–188) and 109 minutes (IQR, 53–178), respectively. This shows that the EDLOS decreased during the pandemic period (*P* < .001).

**Table 2:** Demographic and clinical characteristics of pediatric patients during the control and pandemic periods.

Variables	Control period^∗^ (n = 5172)	Pandemic period^†^ (n = 1422)	*P* value
Age	3.64 ± 3.63	3.66 ± 3.71	.822
Male	2886 (55.80)	799 (56.19)	.794
Patient acuity			<.001
High acuity (KTAS 1, 2, 3)	1773 (34.28)	368 (25.88)	
Low acuity (KTAS 4, 5)	3399 (65.72)	1054 (74.12)	
Reason for visit			<.001
Disease	3585 (69.32)	773 (54.36)	
Trauma	1587 (30.68)	649 (45.64)	
Means of visit			.003
119 ambulances	330 (6.38)	127 (8.93)	.002^‡^
Other ambulances	30 (0.58)	6 (0.42)	.794^‡^
On foot	4812 (93.04)	1289 (90.65)	.006^‡^
Transfer-in	550 (10.63)	151 (10.62)	.987
Outcome			.023
Discharge	4457 (86.18)	1223 (86.01)	
Admission to ward	664 (12.84)	172 (12.10)	
Admission to ICU	28 (0.54)	12 (0.84)	
Transfer-out	21 (0.41)	15 (1.05)	
Death in ED	2 (0.04)	0 (0)	
EDLOS, min	124 (67–188)	109 (53–178)	<.001

The characteristics of the younger adult patients are summarized in Table 3. The mean age of patients who visited during the control period and pandemic period were 38.83 (±13.16) and 39.54 (±12.61), respectively (*P* = .005). Sex, patient acuity, and reason for visit did not differ between the 2 periods. In terms of the means of visit, the proportion of patients using the 119 ambulances increased during the pandemic period (21.51%) compared to the control period (18.46%, *P* < .001), and those who visited on foot decreased during the pandemic period (74.99%) compared to the control period (78.02%, *P* < .001).

**Table 3:** Demographic and clinical characteristics of younger adult patients during the control and pandemic periods.

Variables	Control period^∗^ (n = 8397)	Pandemic period^†^ (n = 3742)	*P* value
Age	38.83 ± 13.16	39.54 ± 12.61	.005
Male	4380 (52.16)	1989 (53.15)	.312
Patient acuity			.378
High acuity (KTAS 1, 2, 3)	3016 (35.92)	1313 (35.09)	
Low acuity (KTAS 4, 5)	5381 (64.08)	2429 (64.91)	
Reason for visit			.133
Disease	5951 (70.87)	2702 (72.21)	
Trauma	2446 (29.13)	1040 (27.79)	
Means of visit			<.001
119 ambulances	1550 (18.46)	805 (21.51)	<.001^‡^
Other ambulances	296 (3.53)	131 (3.50)	1.000^‡^
On foot	6551 (78.02)	2806 (74.99)	<.001^‡^
Transfer-in	852 (10.15)	382 (10.21)	.917
Outcome			.727
Discharge	6617 (78.80)	2982 (79.69)	
Admission to ward	1310 (15.60)	550 (14.70)	
Admission to ICU	333 (3.97)	153 (4.09)	
Transfer-out	115 (1.37)	49 (1.31)	
Death in ED	22 (0.26)	8 (0.21)	
EDLOS, min	143 (87–219)	146 (87–228)	.056

The characteristics of older adult patients are summarized in Table 4. Age, sex, patient acuity, and reason for visit were similar between the 2 periods. However, a similar trend was observed in the older adult patients as in the other 2 groups in terms of the means of visit. The proportion of patients who visited by the 119 ambulances increased during the pandemic period (36.06%) compared to the control period (31.64%, *P* = .001), while the proportion of patients who visited on foot decreased during the pandemic period (50.95%) compared to the control period (54.36%, *P* *=* .027). The EDLOS in the control period was 211 min (IQR, 138–306) and the pandemic period was 226 min (IQR, 143–328), which shows that EDLOS during the pandemic period was increased (*P* = .001).

**Table 4:** Demographic and clinical characteristics of older adult patients during the control and pandemic periods.

Variables	Control period^∗^ (n = 4400)	Pandemic period^†^ (n = 2155)	*P* value
Age	73.62 ± 8.55	74.04 ± 8.78	.065
Male	2249 (51.11)	1141 (52.95)	.163
Patient acuity			.997
High acuity (KTAS 1, 2, 3)	2973 (67.57)	1456 (67.56)	
Low acuity (KTAS 4, 5)	1427 (32.43)	699 (32.44)	
Reason for visit			.997
Disease	3626 (82.41)	1776 (82.41)	
Trauma	774 (17.59)	379 (17.59)	
Means of visit			.002
119 ambulances	1392 (31.64)	777 (36.06)	.001^‡^
Other ambulances	616 (14.00)	280 (12.99)	.518^‡^
On foot	2392 (54.36)	1098 (50.95)	.027^‡^
Transfer-in	953 (21.66)	429 (19.91)	0.102
Outcome			.147
Discharge	8786 (68.66)	3966 (67.25)	
Admission to ward	2784 (21.76)	1363 (23.11)	
Admission to ICU	939 (7.34)	416 (7.05)	
Transfer-out	202 (1.58)	107 (1.81)	
Death in ED	86 (0.67)	45 (0.76)	
EDLOS, min	211 (138–306)	226 (143–328)	.001

### 3.3 Differences of diagnostic codes between control period and pandemic period

Diagnostic codes for pediatric patients between the control period and the pandemic period are summarized in Table 5. There was a significant decrease in certain infectious and parasitic diseases (A00-B99) and respiratory diseases (J00-J99) during the pandemic period compared to the control period. In the control period, the proportions of certain infectious and parasitic diseases (A00-B99) and respiratory diseases (J00-J99) were 29.68% and 14.54%, respectively. Meanwhile, the proportion of these decreased to 20.11% and 4.15%, respectively, during the pandemic period (*P* < .001 and *P* < .001, respectively). Additionally, the proportion of trauma and injuries (S00-T98) significantly increased during the pandemic period (45.99%) compared to the control period (30.74%, *P* < .001). Similar results were observed in the younger adult patients (Table 6). In the control period, the proportions of certain infectious and parasitic diseases (A00-B99) and respiratory diseases (J00-J99) were 8.19% and 9.79%, respectively. However, during the pandemic period, these values decreased to 4.97% and 5.88% respectively (*P* < .001 and *P* < .001, respectively). In older adult patients, no differences in diagnostic codes were found between the control and pandemic periods (Table 7).

**Table 5:** Diagnostic codes based on Korean Standard Classification of Disease-7 of pediatric patients during the control and pandemic periods.

Variables	Control period^∗^ (n = 5172)	Pandemic period^†^ (n = 1422)	*P* value^‡^
Certain infectious and parasitic diseases (A00-B99)	1535 (29.68)	286 (20.11)	<.001
Neoplasms (C00-D48)	6 (0.12)	1 (0.07)	1.000
Hematologic diseases (D50-D89)	13 (0.25)	3 (0.21)	1.000
Endocrine and metabolic diseases (E00-E90)	63 (1.22)	23 (1.62)	.927
Mental and behavioral disorders (F00-F99)	1 (0.02)	1 (0.07)	.994
Neurologic diseases (G00-G99)	26 (0.50)	8 (0.56)	1.000
Cardiac and circulatory diseases (I00-I99)	29 (0.56)	6 (0.42)	1.000
Respiratory diseases (J00-J99)	752 (14.54)	59 (4.15)	<.001
Gastrointestinal diseases (K00-K93)	218 (4.22)	68 (4.78)	.973
Musculoskeletal diseases (M00-M99)	32 (0.62)	16 (1.13)	.416
Trauma and injuries (S00-T98)	1590 (30.74)	654 (45.99)	<.001
Others^∗∗^	907 (17.54)	297 (20.89)	.035

**Table 6:** Diagnostic codes based on Korean Standard Classification of Disease-7 of younger adult patients during the control and pandemic periods.

Variables	Control period^∗^ (n = 8397)	Pandemic period^†^ (n = 3742)	*P* value^‡^
Certain infectious and parasitic diseases (A00-B99)	688 (8.19)	186 (4.97)	<.001
Neoplasms (C00-D48)	148 (1.76)	74 (1.98)	.997
Hematologic diseases (D50-D89)	36 (0.43)	15 (0.40)	1.000
Endocrine and metabolic diseases (E00-E90)	84 (1.00)	40 (1.07)	1.000
Mental and behavioral disorders (F00-F99)	186 (2.22)	91 (2.43)	.999
Neurologic diseases (G00-G99)	225 (2.68)	86 (2.30)	.948
Cardiac and circulatory diseases (I00-I99)	366 (4.36)	170 (4.54)	1.000
Respiratory diseases (J00-J99)	822 (9.79)	220 (5.88)	<.001
Gastrointestinal diseases (K00-K93)	894 (10.65)	382 (10.21)	.999
Musculoskeletal diseases (M00-M99)	238 (2.83)	82 (2.19)	.385
Trauma and injuries (S00-T98)	2443 (29.09)	1042 (27.85)	.854
Others^∗∗^	2267 (27.00)	1354 (36.18)	<.001

**Table 7:** Diagnostic codes based on Korean Standard Classification of Disease-7 of older adult patients during the control and pandemic periods.

Variables	Control period^∗^ (n = 4400)	Pandemic period^†^ (n = 2155)	*P* value^‡^
Certain infectious and parasitic diseases (A00-B99)	211 (4.80)	82 (3.81)	.272
Neoplasms (C00-D48)	296 (6.73)	141 (6.54)	1.000
Hematologic diseases (D50-D89)	31 (0.70)	10 (0.46)	.984
Endocrine and metabolic diseases (E00-E90)	113 (2.57)	61 (2.83)	1.000
Mental and behavioral disorders (F00-F99)	53 (1.20)	23 (1.07)	0.359
Neurologic diseases (G00-G99)	84 (1.91)	26 (1.21)	1.000
Cardiac and circulatory diseases (I00-I99)	526 (11.95)	245 (11.37)	1.000
Respiratory diseases (J00-J99)	531 (12.07)	249 (11.55)	1.000
Gastrointestinal diseases (K00-K93)	506 (11.50)	253 (11.74)	1.000
Musculoskeletal diseases (M00-M99)	100 (2.27)	61 (2.83)	.870
Trauma and injuries (S00-T98)	808 (18.36)	391 (18.14)	1.000
Others^∗∗^	1141 (25.93)	613 (28.45)	.267

## 4 Discussion

The total number of ED patients during the pandemic decreased compared to that in the control period, and similar results have been obtained in studies conducted in other countries.^[7–9,19]^ Pediatric patients had declined more than the other 2 groups during the pandemic period. In addition, whereas the proportion of patients visiting by ambulances increased, the proportion of patients visiting on foot showed a decreasing trend, and the proportion of patients with low acuity and trauma was higher in pediatric patients. Interestingly, the proportion of patients with diagnostic codes for the infectious and respiratory diseases significantly decreased during the pandemic period in pediatric and younger adult patients.

The decrease in ED patients could be attributed to the fear of transmission within medical institutions, as well as the national recommendation that encourages the use of designated screening centers and public health centers instead of the ED. In 2015, there were many cases of transmission in the ED during the MERS outbreak in the Republic of Korea, and this experience may have led to the fear that the infection could spread within the ED.^[10–14]^ It seems that the greater effect occurred in the pediatric population than in the adult population, because of a concern among parents that children are more vulnerable to infections. Similar results have been reported in previous studies.^[12,13]^ According to a study conducted by Paek et al^[12]^ during the 2015 MERS outbreak, the number of adult ED patients decreased by approximately 13% compared to the previous year, and the number of pediatric ED patients decreased by more than 30% compared to the previous year. This seems to be the result of the fear of viral spread among people following cases of transmission within medical institutions. During the SARS outbreak in 2003, there was a reduction in the total number of patients visiting the ED.^[4,20]^ Heiber and Lou^[4]^ reported in their study that the number of ED patients reduced by 21% during the SARS outbreak, and this reduction was particularly notable in infants and toddlers (ages 0–3). Conversely, the results of the study during the influenza H1N1 outbreak demonstrated an increase in the number of patients visiting the ED.^[21,22]^ The study conducted by McDonnell et al^[21]^ showed that there was an increase in the number of ED patients during the 2009 influenza H1N1 outbreak, and this increase was greater in the pediatric population than in the adult population. This could be interpreted as an increase in anxiety about the disease itself as the general public became aware of the influenza H1N1 outbreak and the resulting deaths reported by the media. In particular, it seems that the number of pediatric patients increased due to the increasing anxiety among parents following the reports of deaths in pediatric patients.

Durmuş and Güneysu^[2]^ also studied the effect of COVID-19 on admission to adult ED. Their study was conducted at the Sakarya University Training and Research Hospital in Turkey, and the medical institution was declared a pandemic hospital on March 20, 2020. In their study, the patients who visited the adult ED from March 20 to April 3, 2020, were compared with those who visited during the same period in 2018 and 2019. After the comparison, they discovered that the number of patients admitted in 2020 decreased significantly compared with 2018 and 2019, and a significant decline was observed in the proportion of patients aged 0 to 18 years. Additionally, there was a further decrease in the admissions of female patients and foreign nationals. These results could be because the Sakarya University Training and Research Hospital only treated patients with suspected or confirmed COVID-19 following the March 20, 2020, while patients with no suspicion of COVID-19, such as trauma patients, were transferred to other hospitals, in addition to the closure measures such as closing the border and stopping flights that reduced the number of foreign patients. They reasoned that these closure measures and hospital reorganization resulted in a decrease in the number of patients. However, their study was different from ours in that Inha University Hospital, where we conducted our study, was not reorganized as a COVID-19 pandemic hospital, and patients who were not suspected of COVID-19 also visited the ED. Therefore, in the current study, there was no difference in admission criteria between the control and pandemic periods.

While there was no change in patient acuity between the control and pandemic periods in the younger adult and older adult patients, the proportion of low-acuity patients increased in pediatric patients during the pandemic period. This result may be attributed to an increase in the proportion of trauma patients. During the pandemic period, whereas the proportion of trauma did not change in adult patients, there was an approximately 15% increase in pediatric patients. These results were similar to those of a study conducted during the 2015 MERS outbreak.^[12]^ In addition to anatomical differences compared to adults, children have more difficulty in accurately expressing symptoms following trauma, making it more difficult to identify the injuries clearly.^[23]^ Therefore, accurate diagnosis and treatment are difficult in primary medical institutions, and thus visiting the ED is inevitable even for minor injuries in many cases. Nevertheless, studies conducted in other countries have shown a decrease in the number of trauma patients during the COVID-19 pandemic.^[8,9]^ This difference reflects the characteristics of the South Korean medical system to some extent. There is a high preference for tertiary university hospitals among the public in the Republic of Korea, which leads to problems such as overcrowding, and ED is no exception.^[24–26]^ Thus, the proportion of patients with low acuity or trauma seems to be higher than that of other countries, even during the COVID-19 pandemic.

The proportion of patients who used the 119 ambulance during the pandemic period increased in all 3 groups, whereas the proportion of patients who visited on foot decreased. The use of medical facilities during an infectious disease pandemic occurs when the benefit of using medical facilities outweighs the risk of transmission within a hospital.^[13,27]^ Patients who use an ambulance to visit the ED often have limited mobility or urgent cases, and the severity of illness is usually higher compared to patients who visit the ED on foot.^[28,29]^ Therefore, patients using the 119 ambulance usually require immediate management in the ED, and even if there is a risk of transmission within a hospital, ED visits are inevitable. Because of these characteristics, the proportion of patients using the 119 ambulance may be less affected by the COVID-19 pandemic. In contrast, those who visit the ED on foot have illnesses that are relatively less urgent, and the proportion of this population seemed to have decreased following the concern for transmission within the hospital in comparison to the benefit of using the ED. Additionally, there was no significant difference in the proportion of patients who visited by other ambulances in all 3 groups. This is consistent with the finding that there was no difference in the proportion of transfer-in patients between the control and pandemic periods. This could be interpreted as the proportion of patients requiring higher-level hospital treatment is consistent, even during the pandemic period of an infectious disease. In the same context, the proportion of patients with severe illnesses who visited the ED did not decrease during the MERS and SARS outbreaks.^[12,13,20]^

A previous study showed that the total number of ED patients decreased during the SARS outbreak in 2003, but the number of patients with upper respiratory infections increased.^[4]^ In addition, another study showed a marked increase in the number of patients with symptoms of upper respiratory infection during the influenza H1N1 outbreak.^[30]^ Therefore, we predicted that the number of patients with infectious and respiratory diseases during the pandemic period would be higher than that in the control period. However, contrary to this prediction, the pediatric and younger adult patients in this study showed a significant decrease in infectious and respiratory disease cases. These results seem to reflect the national recommendations that encourage social distancing, wearing of face masks and hand hygiene. The government of the Republic of Korea has recommended wearing of face masks and hand hygiene since the beginning of the COVID-19 pandemic, which have been proven to be effective in preventing the transmission of infectious diseases such as influenza-like illness and COVID-19.^[31–33]^ Although these national recommendations were introduced specifically to stop the spread of COVID-19, wearing face masks also helped prevent other droplet-transmitted infectious diseases. Previous studies have shown decreased influenza activity during the COVID-19 pandemic.^[34,35]^ The study by Lee et al^[34]^ reported that the national response of the Republic of Korea for the prevention of COVID-19 resulted in a marked reduction in seasonal influenza activity. The significant reduction in respiratory infectious diseases during the pandemic period of this study can also be interpreted in the same context, and these national recommendations are expected to have a preventive effect in the event of other respiratory infectious diseases, including influenza. In addition, this result may be attributed to the mild patients who did not visit the ED. As mentioned earlier, the government of the Republic of Korea recommended the use of designated screening centers and public health centers instead of the ED. While patients with severe respiratory infectious symptoms have visited the ED, patients with mild symptoms may have also visited the screening clinics and public health centers, rather than the ED. Consequently, the reduction of respiratory infectious diseases caused by the national recommendations, such as mask wearing, and the non-visiting of the ED by mild patients, may have contributed to the results.

To the best of our knowledge, this is the first study to analyze the effects of the COVID-19 pandemic on the use of ED in the Republic of Korea. It is meaningful in that we compared not only the demographic and clinical characteristics, but also the differences in diagnostic codes. It is difficult to determine the impact of COVID-19 on the ED in all countries. To assess this more clearly, further studies using international data are required. Given the need for quick response of emergency medical facilities in the event of a pandemic of infectious diseases such as COVID-19, these studies will provide a blueprint for the proper operation of ED in the future when there is an outbreak of another infectious disease. In this study, the COVID-19 pandemic has resulted in a decrease in the total number of ED patients, and this decrease was more pronounced in pediatric patients. For efficient ED operation in a pandemic situation, the redeployment of medical personnel may be considered. Meanwhile, the proportion of patients using ambulances has increased. This suggests that patients who require immediate treatment in the ED and those who require higher-level hospital treatment are consistent despite the pandemic of infectious diseases. Therefore, even during the pandemic period, the role of the ED in providing proper treatment to urgent patients should continue. In order for the ED to maintain this role, efforts to prevent in-hospital transmissions are also needed. This includes preparing facilities to isolate infectious patients and wearing protective equipment to prevent transmission through medical personnel. Additionally, preventive measures, such as mask wearing, hand hygiene, and social distancing, should be swiftly taken. These measures can also be expected to reduce the number of patients with infectious and respiratory diseases.

This study has several limitations. First, the results cannot be generalized to all medical institutions as this is a single-center, retrospective observational study. Second, this study was conducted under the assumption that there are no other factors that affected ED visits other than COVID-19 during the pandemic period. Therefore, the proposition that other factors in addition to COVID-19, could have affected the results during the study period cannot be ruled out. Third, the KTAS used in this study may not accurately reflect the severity of the disease because of the subjectivity of the medical personnel performing the triage. However, these errors would have been minimized as only qualified personnel who completed the relevant training participated in the KTAS classification.

In conclusion, the total number of patients visiting the ED during the COVID-19 pandemic decreased. This decrease was more pronounced in pediatric patients, but the proportion of patients using ambulances increased in all age groups. In particular, the number of patients diagnosed with certain infectious or parasitic diseases (A00-B99) and respiratory diseases (J00-J99) was greatly reduced, which could be attributed to national recommendations such as wearing face masks, hand hygiene, and social distancing.

## Acknowledgments

The authors thank all the healthcare workers who are working on COVID-19 treatment. And we would like to thank Editage for English language editing.

## Author contributions

**Conceptualization:** Soo Kang, Jin Hui Paik.

**Data curation:** Soo Kang, Young Ho Seo, Young Ju Suh.

**Formal analysis:** Soo Kang, Tae Kyu Ahn.

**Funding acquisition:** Jin Hui Paik.

**Investigation:** Jin Hui Paik, Young Ho Seo.

**Methodology:** Soo Kang.

**Project administration:** Soo Kang, Jin Hui Paik.

**Resources:** Soo Kang.

**Software:** Soo Kang.

**Supervision:** Jin Hui Paik, Young Ju Suh.

**Validation:** Young Ju Suh.

**Writing – original draft:** Tae Kyu Ahn, Young Ho Seo.

**Writing – review & editing:** Tae Kyu Ahn, Young Ho Seo.
